# Predicting adverse events after thoracic endovascular aortic repair for patients with type B aortic dissection

**DOI:** 10.1038/s41598-024-58106-7

**Published:** 2024-04-05

**Authors:** Mengyang Kang, You Li, Yiman Zhang, Yang Zhao, Yan Meng, Junbo Zhang, Hongyan Tian

**Affiliations:** Department of Peripheral Vascular Diseases, The First Affiliated Hospital of Xi’an Jiaotong University, No. 277, Yanta West Road, Xi’an, 710061 Shaanxi China

**Keywords:** Type B aortic dissection, Computed tomography angiography, Thoracic endovascular aortic repair, Adverse events, Prediction model, Aortic diseases, Risk factors

## Abstract

The potential of adverse events (AEs) after thoracic endovascular aortic repair (TEVAR) in patients with type B aortic dissection (TBAD) has been reported. To avoid the occurrence of AEs, it is important to recognize high-risk population for prevention in advance. The data of 261 patients with TBAD who received TEVAR between June 2017 and June 2021 at our medical center were retrospectively reviewed. After the implementation of exclusion criteria, 172 patients were finally included, and after 2.8 years (range from 1 day to 5.8 years) of follow up, they were divided into AEs (n = 41) and non-AEs (n = 131) groups. We identified the predictors of AEs, and a prediction model was constructed to calculate the specific risk of postoperative AEs at 1, 2, and 3 years, and to stratify patients into high-risk (n = 78) and low-risk (n = 94) group. The prediction model included seven predictors: Age > 75 years, Lower extremity malperfusion (LEM), NT-proBNP > 330 pg/ml, None distal tear, the ratio between the diameter of the ascending aorta and descending aorta (A/D ratio) > 1.2, the ratio of the area of the false lumen to the total aorta (FL ratio) > 64%, and acute TEVAR, which exhibited excellent predictive accuracy performance and discriminatory ability with C statistic of 82.3% (95% CI 77.3–89.2%). The prediction model was contributed to identify high-risk patients of postoperative AEs, which may serve to achievement of personalized treatment and follow-up plans for patients.

## Introduction

Thoracic endovascular aortic repair (TEVAR) has largely supplanted open surgical repair as an important treatment option for type B aortic dissection (TBAD) given lower morbidity and mortality rates^1^, which aims to cover the proximal entry tear, expand the true lumen (TL) while decreasing the size of the false lumen (FL), avoid perfusion of the FL, and achieve aortic remodeling^2,3^. However, some potentially postoperative disastrous adverse events (AEs) still may occur^4,5^, such as retrograde type A aortic dissection (RTAD)^6^, paraplegia, neurological events^7^, endoleak and stent graft-induced new entry tear (SINE)^8^, which represents a major health and socioeconomic burden globally. Thus, early identification of patients at the high-risk of postoperative AEs is necessary.

Computed tomography angiography (CTA) excels in the diagnosis and follow-up of patients with aortic dissection. Previous studies have confirmed that CTA images association with short- and long-term outcomes in patients with TBAD^9,10^, but few studies have used imaging morphological parameters to predict postoperative AEs in patients with TBAD. Therefore, this study developed a new comprehensive prediction model, including the clinical characteristics, laboratory test results, preoperative morphological parameters, and intraoperative conditions, to identify the high-risk population of AEs following TEVAR accurately and rapidly in patients with TBAD. The prediction model may contribute to enhance personalized follow-up programs and prevent the occurrence of post-operative AEs.

## Methods

### Study participants

**Figure 1:**
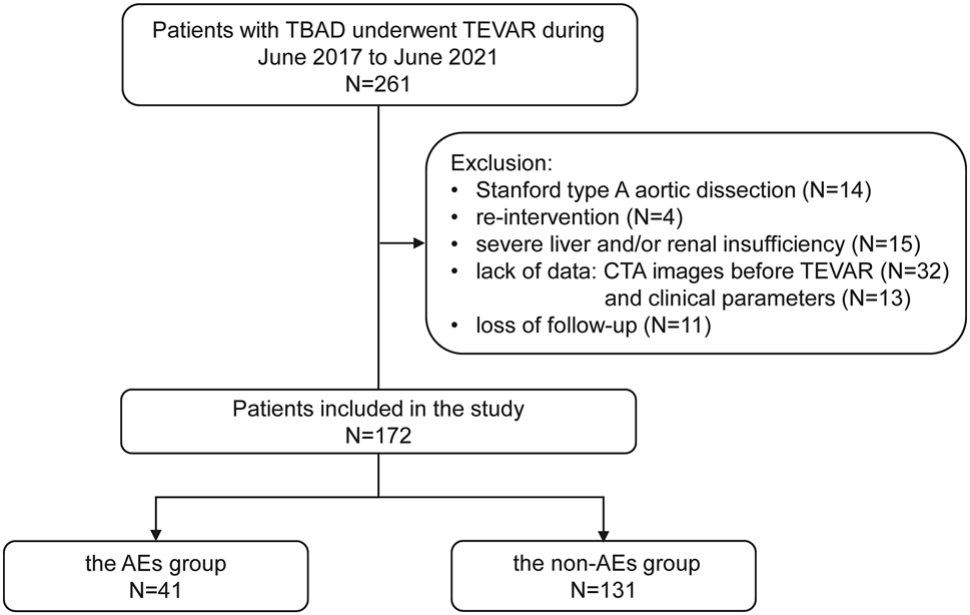
A consort diagram of the study population. TBAD, type B aortic dissection; TEVAR: thoracic endovascular aortic dissection; CTA: computed tomography angiography; AEs: adverse events.

All patients treated at our medical center for TBAD (n = 426) and underwent TEVAR (n = 261) from June 2017 to June 2021 were consider eligible for our study. The exclusion criteria of this study included patients with type A aortic dissection (TAAD), previous TEVAR, severe liver and/or renal disease. Patients with missing data and loss of follow-up were also excluded. Finally, a cohort of 172 were enrolled in our final analysis (Fig. 1). The clinical characteristics, laboratory test results, CTA imaging parameters, intraoperative conditions, and stent-graft information were collected from all 172 subjects.

### Data collection and definition

We collected the general and clinical characteristics, laboratory test results from the hospital E-cases. The CTA imaging data were collected by the department of information management in our hospital. The intraoperative conditions, and stent-graft information were extracted from the operation records. Clinical classification of TBAD is based on the duration from symptom onset to admission: acute phase (≤ 14 days), subacute phase (15–90 days), and chronic phase (> 90 days). Lower extremity malperfusion (LEM) was defined as a nonpalpable femoral artery pulse and at least one of the following additional findings: pain, decreased sensation or motor function, limb discoloration, or computed tomography demonstration of arterial obstruction of the affected limb/limbs by the dissection. Traumatic aortic injury (TAI) refers to TBAD caused by sudden trauma, such as moto vehicle accident, falls, and other external forces. (other risk factors for aortic dissection have been excluded). History of cardiac operation was defined as previous cardiac intervention or surgery, including percutaneous coronary intervention (PCI) or coronary artery bypass grafting (CABG). Individuals who drank more than 20 g per day were defined as alcohol drinkers. Smokers were defined as those who had regularly smoking in the previous six months. Tapered stent-graft (TSG) refers to a stent characterized by a larger proximal diameter compared to its distal diameter, while a stent with equal proximal and distal diameter is classified as a non-TSG. Post-implantation syndrome (PIS) was defined as fever > 38℃, white blood cell (WBC) > 12.0/ nl and C-reaction protein (CRP) > 10 mg/dl within 72 hours after TEVAR despite negative blood culture results.

### CTA imaging data

All imaging data were exported and retrieved from the image repository of the Department of Information management in the Frist Affiliated Hospital of Xi’an Jiaotong University, and then transferred to a dedicated workstation (Endosize, version 3.1.42; Therenva SAS, Rennes, France). Two experienced cardiovascular specialists reviewed the consistency of CTA images obtained at the time of the hospitalization. If multiple CTA images of comparable diagnostic quality were acquired prior to the TEVAR procedure, the most recent one before TEVAR was used to evaluation.

### Aortic measurements

The preoperative CTA images were transferred to a dedicated workstation (Endosize, version 3.1.42; Therenva SAS, Rennes, France) for three-dimensional reconstruction of the aorta lumen along its central line. Automated location, which determines boundaries around voxels with a similar intensity, was used and manually adjusted to select total aorta lumen, true lumen (TL) or false lumen (FL). Subsequently, the diameters of lumen in every segment were calculated automatically. Measurements were performed by two experienced cardiovascular specialists and reviewed by another specialist, both of whom unaware of patient information.

The definition and calculation method of morphological indicators in this study are as follows: (Supplement Fig. S1) (1) the measurement of the elliptical aortic lumen diameter is based on the long diameter (LD) unless it exceeds 5% of the short diameter (SD), in which case it should be calculated as the average of LD and SD. (2) the distance from primary entry tear to LSA: for primary entry tear located at he the inner curve of aorta, the distance should be measured as internal length; for those located at the outer curve of aorta, it should be measured as external length; and if the primary entry tear is located at the anterior or posterior of aorta, center line length is used for measurement. (3) The length of stent-graft was measured along the outer curve of the aorta. (4) A/D ratio: the ratio of TL diameters between the ascending aorta and descending aorta, measured at the level of pulmonary trunk bifurcation. (5) FL ratio: the maximum ratio of FL area to the total lumen area. To better illustrate the measurement methods of the above indicators, we have drawn a schematic diagram (Supplement Fig. S2). The time required for a complete evaluation of morphological features from a single CTA dataset is approximately 30 min.

### Procedure principles

The TEVAR procedures were conducted by operators with a minimum of 5 years of interventional experience, and our procedure principles are as follow: (i) the primary entry tear must be completely excluded, and the patency of the distal lumen should be confirmed following the implantation of stent-grafts; (ii) for patients with TBAD involving the LSA, revascularization techniques should be recommended to achieve complete exclusion of the primary entry tear while preserving the blood supply of LSA; (iii) based on individual anatomical characteristics of the aorta and LSA, an appropriate revascularization technique for the LSA should be selected.

### Endpoints and follow-up 

Patients were divided into the AEs group and non-AEs group based on the occurrence of postoperative AEs. The endpoints time of follow-up were defined as the time at which patients initially experienced an AE following TEVAR, or in the absence of any AEs, it was the time of the last telephone follow-up, outpatient visit or CTA examination. AEs were defined as follows: aortic-related mortality, aortic rupture, retrograde type A aortic dissection (RTAD), type I endoleak, stent graft-induced new entry tear (SINE), paraplegia, neurological events, limb or visceral ischemia, stent-graft infection, post-implantation syndrome (PIS) and aortic branch vascular stenosis. Exclude similar conditions from other diseases, such as acute coronary syndrome, advanced malignancy, etc. Routine follow-up CTA images were obtained at 3, 6, and 12 months, and annually thereafter, but actual follow-up schedules vary between individuals.

### Statistical analysis

Descriptive statistics were reported as mean ± standard deviation (SD) or median and interquartile range (IQR). Continuous variables were compared using the student’s t-test, while the Mann–Whitney U test was employed in the absence of a normal distribution. The association between the postoperative AEs and categorical variables using the χ^2^ test or Fisher exact test. The end points were analyzed descriptively using Kaplan–Meier estimates, and inferentially by univariate and multivariate Cox regression analysis. Accuracy evaluation involved constructing receiving operating characteristic (ROC) curve. Furthermore, the time-dependent ROC curves and calibration curves were employed to assess the predictive ability of the nomogram of the prediction model. Statistical analyses were performed using R software version 3.6.3. All statistical assessments were two-tailed and considered significantly different at P < 0.05.

### Ethics approval

This retrospective observational cohort study was approved by the Ethics Committee of the First Affiliated Hospital of Xi’an Jiaotong University (No: XJTU1AF2022LSK-234). Verbal informed consent was obtained from the patient(s) to published their anonymized clinical information and CTA images in this article.

## Results

### Patient information

We identified a total of 172 patients and described the baseline characteristics (Table 1), the baseline CTA imaging characteristics, the intraoperative conditions and stent-graft information (Table 2). 134 men (77.9%), and 38 women (22.1%), with a mean age of 55 ± 11.5 years (range from 30 to 83 years). The median follow-up time was 2.8 (1.5–3.8) years. 140 (81.4%) of patients received TEVAR in the acute phase, and the remaining (18.6%) underwent TEVAR in the subacute phase. AEs were observed in 41 (23.84%) of patients: aortic related mortality developed in 10 (5.81%) patients, type I endoleak observed in 8 (4.65%) patients, RTAD occurred in 4 (2.33%) patients and one fatality, limb ischemia developed in 4 (2.33%) patients, PIS observed in 4 (2.33%) patients, LSA stenosis detected in 3 (1.74%) patients, stroke diagnosed in 2 (1.16%) patients, 1 (0.58%) patient developed paraplegia, and 1 (0.58%) patient presented with aortic rupture. The duration of AEs following TEVAR in patients of our study is shown in Supplement Fig. S3.

**Table 1:** Baseline characteristics.

Variables	All patients (n = 172)	the non-AEs group (n = 131)	The AEs group (n = 41)	*P*
Follow-up time, years	2.8 (1.5–3.8)	3.1 (2.1–4.0)	0.2(0.02–1.0)	< 0.001
Age(years)	55.5 ± 11.5	55.5 ± 11.1	55.6 ± 13.0	0.968
Age≥ 74 years, n (%)	9 (5.23%)	3 (2.29%)	6 (14.6%)	**0.006**
Male, n (%)	134 (77.9%)	100 (76.3%)	34 (82.9%)	0.502
SBP, mmHg	163 ± 29.6	164 ± 30.3	159 ± 27.3	0.295
DBP, mmHg	94.6 ± 18.8	95.2 ± 19.4	92.5 ± 17.1	0.404
HR, time/min	79.7 ± 13.6	79.8 ± 13.8	79.1 ± 13.2	0.760
Sudden chest pain, n (%)	129 (75.0%)	102 (77.9%)	27 (65.9%)	0.179
Myocardial ischemia, n (%)	69 (40.1%)	55 (42.0%)	14 (34.1%)	0.477
Pericardial effusion, n (%)	12 (6.98%)	9 (6.87%)	3 (7.32%)	1.000
Pleural effusion, n (%)	56 (32.6%)	43 (32.8%)	13 (31.7%)	1.000
LEM, n (%)	20 (11.6%)	10 (7.63%)	10 (24.4%)	0.009
Hypertension, n (%)	126 (73.3%)	101 (77.1%)	25 (61.0%)	0.067
History of TAA, n (%)	4 (2.33%)	2 (1.53%)	2 (4.88%)	0.241
Marfan syndrome, n (%)	2 (1.16%)	1 (0.76%)	1 (2.44%)	0.421
TAI, n (%)	6 (3.49%)	5 (3.82%)	1 (2.44%)	1.000
History of cardiac operation, n (%)	4 (2.33%)	2 (1.53%)	2 (4.88%)	0.241
Diabetes, n (%)	7 (4.07%)	6 (4.58%)	1 (2.44%)	1.000
CAD, n (%)	20 (11.6%)	16 (12.2%)	4 (9.76%)	0.786
Stroke, n (%)	18 (10.5%)	12 (9.16%)	6 (14.6%)	0.380
COPD, n (%)	15 (8.72%)	11 (8.40%)	4 (9.76%)	0.757
Cancer, n (%)	5 (2.91%)	3 (2.29%)	2 (4.88%)	0.594
Smoker, n (%)	89 (51.7%)	63 (48.1%)	26 (63.4%)	0.125
Drinker, n (%)	17 (9.88%)	14 (10.7%)	3 (7.32%)	0.765
Hb, g/L	134 ± 21.6	135 ± 20.6	132 ± 24.5	0.541
PLT, × 10^9^/L	180 ± 62.6	181 ± 62.3	176 ± 64.2	0.612
WBC, × 10^9^/L	9.96 ± 3.46	10.0 ± 3.49	9.76 ± 3.42	0.677
NLR, %	7.2 (4.1–12.5)	7.0 (4.0–11.5)	7.9 (5.0–13.8)	0.426
CRP, mg/L	10.0 (10.0–43.5)	10.0 (10.0–43.6)	10.0 (10.0–31.8)	0.899
ALT, U/L	20.0 (13.0–33.8)	19.0 (13.0–34.0)	23.0 (15.5–34.0)	0.353
Scr, μmol/L	65.0 (53.3–88.8)	63.0 (52.0–80.0)	79.0 (61.5–96.5)	**0.004**
D-dimer, mg/L	2.7 (1.4–5.5)	2.7 (1.5–5.5)	2.7 (1.1–7.0)	0.901
NT-proBNP, pg/mL	219.1 (97.3–485.7)	198.4 (88.6–399.6)	368.0 (111.0–870.1)	**0.021**
NT-proBNP> 330 pg/mL, n(%)	59 (34.3%)	37 (28.2%)	22 (53.7%)	**0.005**
CK, U/L	70.5 (45.0–122.0)	67.0 (44.0–113.0)	87.0 (49.0–162.0)	0.233

Values are expressed as mean ± SD or n (%), SD, standard deviation.

Significant values are in bold.

SBP, systolic blood pressure; DBP, diastolic blood pressure; HR: heart rate; LEM, lower extremity malperfusion; TAA, thoracic aortic aneurysm; TAI, traumatic aortic injury; CAD, coronary artery disease; COPD, chronic obstructive pulmonary disease; Hb, hemoglobin; PLT, platelet; WBC, white blood cell; NLR, neutrophil-to-lymphocyte ratio; CRP, C-reactive protein; ALT, alanine aminotransferase; Scr, serum creatinine; NT-proBNP: N-terminal pro-B-type natriuretic peptide; CK, creatine kinase.

**Table 2:** Baseline CTA imaging characteristics, the intraoperative conditions and stent-graft information.

Variables	All patients (n = 172)	the non-AEs group (n = 131)	The AEs group (n = 41)	*P*
Baseline CTA imaging characteristics				
The aortic diameter of the proximal end of the LSA, mm	29.1 ± 2.61	29.3 ± 2.47	28.6 ± 2.97	0.198
The aortic diameter of the tracheal bifurcation, mm	36.0 ± 6.43	35.8 ± 6.42	36.5 ± 6.49	0.529
The aortic diameter of the tracheal bifurcation> 40 mm, n (%)	37 (21.5%)	23 (17.6%)	14 (34.1%)	**0.042**
The ascending aortic diameter, mm	37.8 ± 3.77	38.0 ± 3.73	37.4 ± 3.92	0.458
The descending aortic diameter, mm	34.9 ± 7.19	34.8 ± 7.06	35.4 ± 7.69	0.623
The A/D ratio	1.1 (1.0–1.24)	1.08 (1.0–1.2)	1.2 (1.0–1.4)	** < 0.001**
The A/D ratio> 1.2, n (%)	56 (32.6%)	33 (25.2%)	23 (56.1%)	** < 0.001**
The maximum descending aortic diameter, mm	41.8 ± 8.31	41.4 ± 7.92	43.0 ± 9.46	0.336
The diameter of the primary entry tear, mm	10.0 (5.5–15.4)	9.7 (5.2–14.0)	14 (6.7–19.5)	0.008
The diameter of the primary entry tear> 12.8 mm, n (%)	65 (37.8%)	40 (30.5%)	25 (61.0%)	**0.001**
The location of the primary entry tear (inner curvature), n (%)	134 (77.9%)	101 (77.1%)	33 (80.5%)	0.810
The median distance from the primary entry tear to the LSA, mm	23.0 (15.0–35.85)	22.8 (15.9–34.6)	23.3 (12.6–41.2)	0.894
None distal tear, n (%)	60 (34.9%)	34 (26.0%)	26 (63.4%)	** < 0.001**
The length of the thoracic aorta, mm	302.0 (274.3–326.5)	303.0 (275.0–322.0)	298.0 (271.0–332.0)	0.651
The length of stent graft, mm	200.0 (160.0–226.3)	200.0 (160.0–215.0)	200.0 (180.0–265.0)	0.172
Maximum area of the total aortic lumen, mm^2^	942.2 (732.8–1244.4)	938.0 (731.2–1170.3)	965.2 (744.5–1608.5)	0.449
Maximum area of the false lumen, mm^2^	620.8 (424.7–916.4)	589.6 (415.5–865.6)	648.1 (524.9–1194.3)	**0.034**
The FL ratio, %	30.6 (19.6–44.9)	67.4 (51.9–78.7)	76.7 (66.2–84.2)	**0.002**
The FL ratio> 64%, n (%)	110 (64.0%)	74 (56.5%)	36 (87.8%)	**0.001**
The branch arteries involvement, n (%)	95 (55.2%)	71 (54.2%)	24 (58.5%)	0.758
The intraoperative conditions and stent-graft information				
Acute TEVAR, n (%)	140 (81.4%)	106 (80.9%)	34 (82.9%)	0.953
Stent graft passage (LFA), n (%)	37 (21.5%)	25 (19.1%)	12 (29.3%)	0.243
LSA coverage, n (%)				
0%	136 (79.1%)	106 (80.9%)	30 (73.2%)	0.153
25%	3 (1.74%)	2 (1.53%)	1 (2.44%)	
50%	23 (13.4%)	17 (13.0%)	6 (14.6%)	
75%	2 (1.16%)	0 (0.00%)	2 (4.88%)	
100%	8 (4.65%)	6 (4.58%)	2 (4.88%)	
LSA revascularization techniques				
Branched stent-graft, n (%)	36 (20.9%)	31 (23.7%)	5 (12.2%)	0.175
Chimney technique, n (%)	41 (23.8%)	30 (22.9%)	11 (26.8%)	0.760
Fenestration, n (%)	5 (2.91%)	3 (2.29%)	2 (4.88%)	0.594
Bank of stent-graft, n (%)				
1	54 (31.4%)	38 (29.0%)	16 (39.0%)	**0.026**
2	106 (61.6%)	87 (66.4%)	19 (46.3%)	
3	12 (6.98%)	6 (4.58%)	6 (14.6%)	
The distal covered stenting, n (%)	58 (33.7%)	40 (30.5%)	18 (43.9%)	0.164
TSG, n (%)	127 (73.8%)	98 (74.8%)	29 (70.7%)	0.753
Operator, n (%)				
A	61 (35.5%)	49 (37.4%)	12 (29.3%)	0.634
B	72 (41.9%)	53 (40.5%)	19 (46.3%)	
C	39 (22.7%)	29 (22.1%)	10 (24.4%)	
Intraoperative endoleak, n (%)	19 (11.0%)	12 (9.16%)	7 (17.1%)	0.163

Values are expressed as mean ± SD or n (%), SD, standard deviation.

Significant values are in bold.

CTA, computed tomography angiography; LSA, left subclavian artery; A/D ratio, the ratio of TL diameters between the ascending and descending aorta, measured at level of pulmonary trunk bifurcation; FL, false lumen. TEVAR; thoracic endovascular aortic repair; LFA, left femoral artery; TSG; tapered stent-graft.

### Prediction model development

Univariate regression analysis was conducted on 62 variables, including the general and clinical characteristics, laboratory test results, preoperative morphological parameters of CTA images, intraoperative conditions, and stent-graft information (Supplement Tables S1, S2). We found 11 variables significantly associated with AEs after TEVAR in patients with TBAD (P < 0.05). Based on the results of univariate analysis, 11 variables were included in the multivariate regression analysis. 7 variables related to the postoperative AEs were selected by backward stepwise elimination. The 7 significant predictors of postoperative AEs are as follow: Age > 74 years (HR 3.494, 95% CI 1.336–9.139, *P* = 0.01), LEM (HR 2.690, 95% CI 1.278–5.673, *P* = 0.009), NT-proBNP > 330 pg/ml (HR 2.360, 95% CI 1.214–4.591, *P* = 0.011), None distal tear (HR 4.511, 95% CI 2.253–9.029, *P* < 0.001), A/D ratio > 1.2 (HR 2.014, 95%CI 1.041–3.895, *P* = 0.038), FL ratio > 64% (HR 4.245, 95% CI 1.572–11.460, *P* = 0.004), Acute TEVAR (HR 2.492, 95% CI 1.037–5.985, *P* = 0.041). The hazard ratios with confidence intervals and coefficients are presented in Table 3, and the forest plot is provided in Supplement Fig S4. We included these 7 variates into a prediction model.

**Table 3:** Multivariate analyses for predictors of postoperative AEs in patients with TBAD.

Predictors	Regression Coefficient	HR (95% CI)	*P*
Age > 74 years	1.251	3.494 (1.336–9.139)	0.010
LEM, n (%)	0.991	2.690 (1.278–5.673)	0.009
NT-proBNP > 330 pg/ml, n (%)	0.859	2.360 (1.214–4.591)	0.011
None distal tear, n (%)	1.506	4.511 (2.253–9.029)	< 0.001
A/D ratio > 1.2, n (%)	0.700	2.014 (1.041–3.895)	0.038
FL ratio > 64%, n (%)	1.446	4.245 (1.572–11.460)	0.004
Acute TEVAR, n (%)	0.913	2.492 (1.037–5.985)	0.041

Selection of variables was based on backward stepwise elimination.

HR, hazard ratio; CI, confidence interval; LEM, lower extremity malperfusion; NT-proBNP: N-terminal pro-B-type natriuretic peptide; A/D ratio, the ratio of TL diameter of the ascending and descending aorta; FL, false lumen; TEVAR, thoracic endovascular aortic repair.

**Figure 2:**
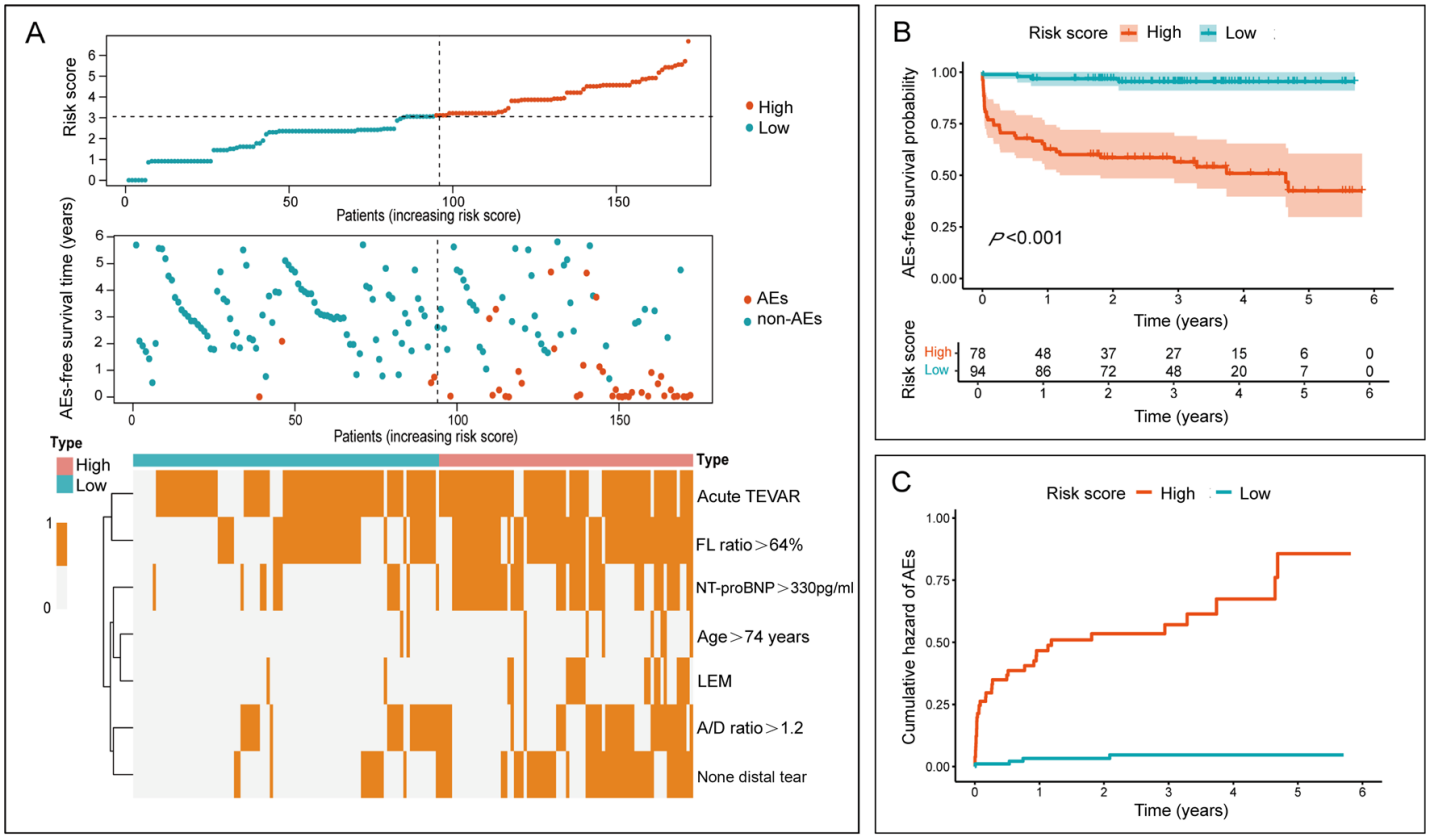
The risk score, AEs-free survival, and incidence characteristics of patients in the study. (**A**) The distributed characteristics (risk score, AEs-free survival time and status) and heatmap of the patients with TBAD underwent TEVAR. The dotted lines indicated the optimal cut-off value between the low- and high-risk group; (**B**) Kaplan–Meier curves stratified by optimal cut-off value of the score on the prediction model with the confidence limits as a colored shaded area; (**C**) The cumulative incidence curves of AEs using cumulative incidence method in each group.

The regression equation for the prediction model: ln[h(t, X)/h_0_(t)] = 1.251 × Age > 74 years + 0.991 × LEM + 0.859 × NT-proBNP > 330 pg/ml + 1.506 × None distal tear + 0.700 × A/D ratio > 1.2 + 1.446 × FL ratio > 64% + 0.913 × Acute TEVAR. We used the values of β coefficient and reference value of each variable of the above predictors to calculate the individual risk score. The score of patients ranged from 0 to 6.7 points. The ROC curve analysis of the prediction model showed that an optimal cut-off score of 3.09 points, with a sensitivity of 90.2% and a specificity of 68.7% (AUC = 0.865, *P* < 0.05). We stratified individuals into two groups on the optimal cut-off value: the low-risk (n = 94) and the high-risk (n = 78) for postoperative AEs. The patients classified as the high-risk group exhibited a significant higher risk for developing AEs compared to those in the low-risk group (HR 13.976, 95% CI 4.976–39.249, *P* < 0.000). Compared with patients in the low-risk group, patients in the high-risk group had higher risk score and more risk factors for postoperative AEs (Fig. 2A). The cumulative rate of AEs-free survival in the low-risk group was significantly higher than that in the high-risk group (*P* < 0.001) (Fig. 2B). The cumulative incidence of postoperative AEs in the high-risk group was significantly higher than that in the low-risk group (*P* < 0.001) (Fig. 2C).

### Prediction model performance and internal validation

**Figure 3:**
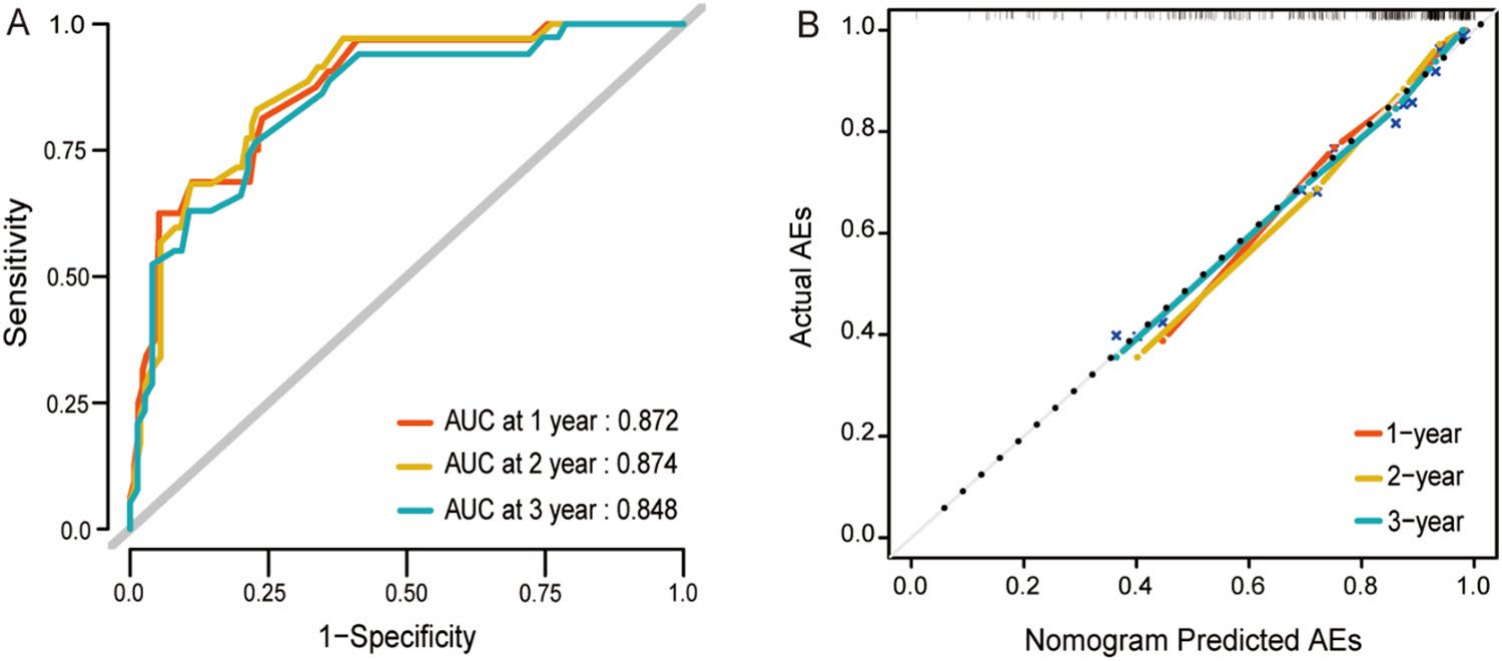
Assessment of the predictive ability of the nomogram. (**A**) Time-dependent ROC curves for evaluating the model’s discrimination performance of the 1-,2- and 3-year AEs-free survival outcome; area under the curves (AUC) was 0.872, 0.874 and 0.848, respectively. (**B**) Calibration curves for the prediction model. The curves depict the calibration of the nomogram in terms of agreement between predicted risks and actual outcomes of AEs. The number of bootstraps that were used was 1000. The x and y axes represent the predicted risk and actual outcome, respectively. The black dotted line indicates perfect prediction by an ideal model.

The C statistic of the model was 82.3% (95% CI: 77.3–89.2%). Figure 3A showed the time-dependent ROC curves of the model. The prediction model has a quite good predictive value for the occurrence of AEs after TEVAR at 1-year (AUC = 0.872), 2-years (AUC = 0.874) and 3 years (AUC = 0.848) in patients with TBAD. The actual AEs and nomogram-predicted AEs matched well at 1-, 2- and 3-year, as shown by the calibration curves (Fig. 3B). To validate the accuracy of the prediction model, we used a bootstrap approach for internal validation. The bootstrap samples demonstrated a similar predictive value and accuracy for the postoperative AEs at 1-year (AUC = 0.829), 2-years (AUC = 0.831) and 3 years (AUC = 0.810).

### Model presentation

**Figure 4:**
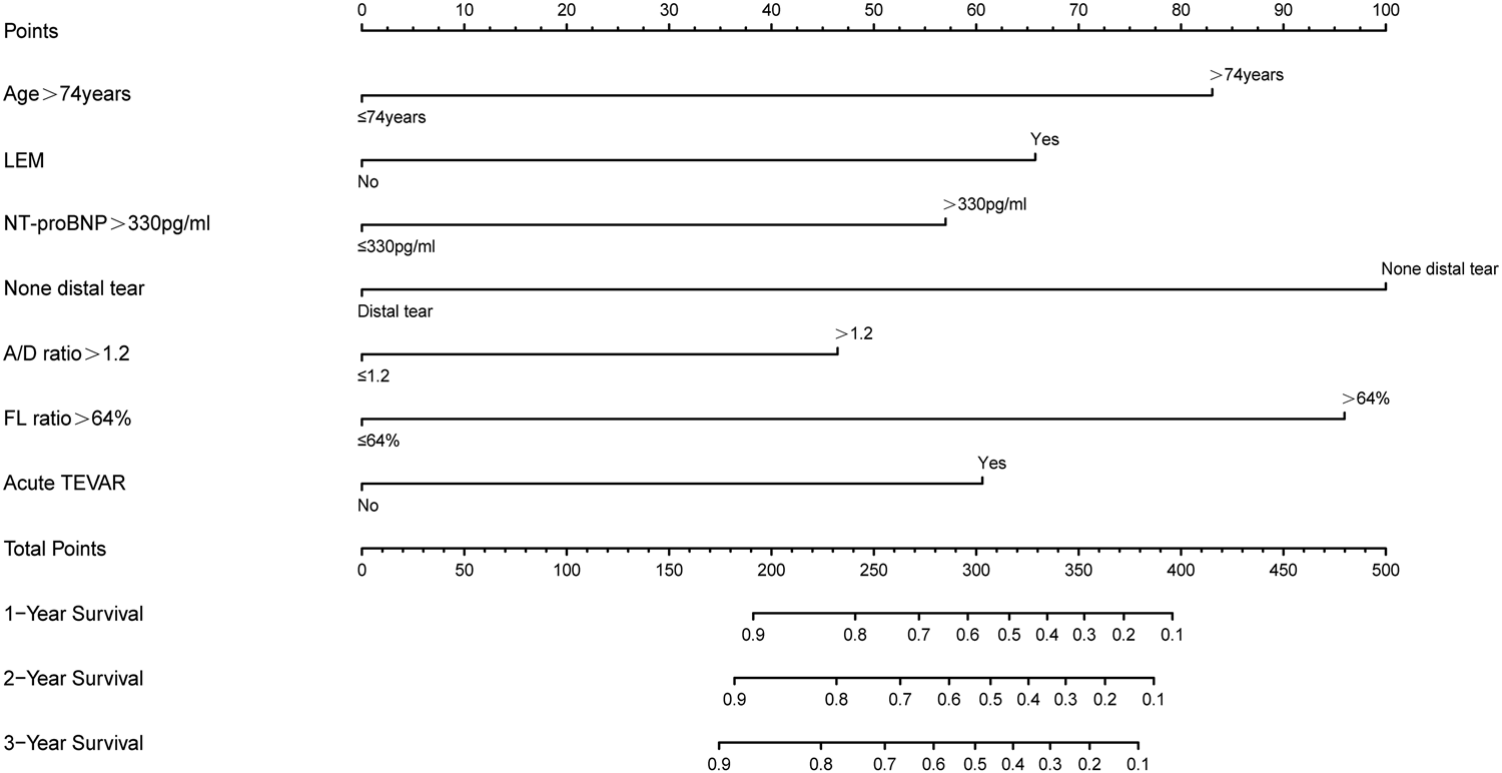
Nomogram for predicting 1-, 2- and 3-year AEs-free survival of patients with TBAD following TEVAR.

**Figure 5:**
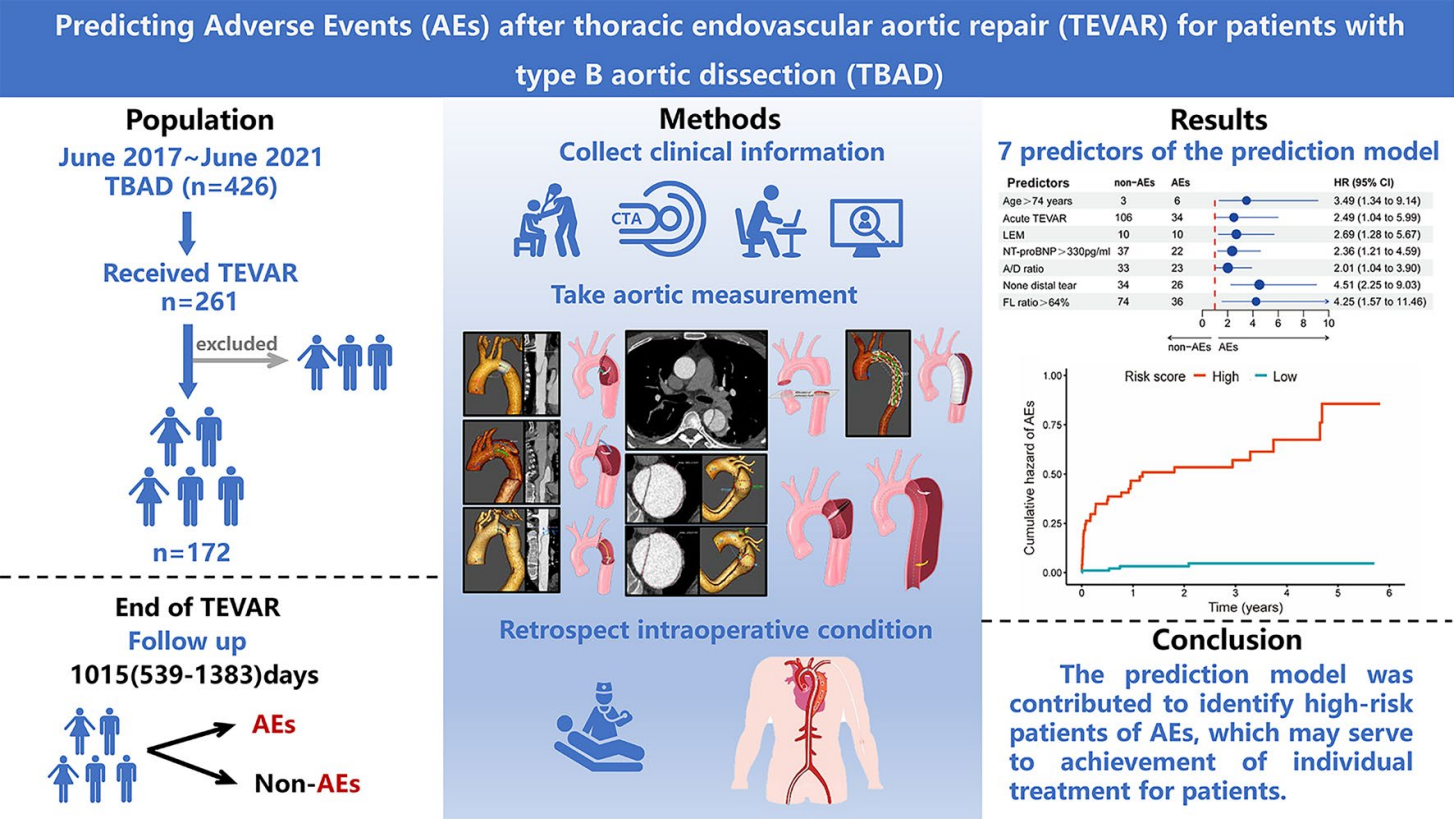
An overview of our study.

Given the absence of the prediction models included CTA imaging parameters, but it is notable that they performed well in preoperatively predicting postoperative AEs. The study aims to develop a novel prediction model that can effectively identify patients at risk of postoperative AEs. Our model allows early identification of patients with TBAD at the high-risk of postoperative AEs in advance. Finally, to generate and validate the prediction model that could be translated to the clinic, we developed a nomogram for better visualization (Fig. 4). To enhance the clarity of our study, we have prepared an overview (Fig. 5).

## Discussion

The findings of this study can be summarized as follows: (i) Age > 74 years, LEM, NT-proBNP > 330 pg/ml, None distal tear, A/D ratio > 1.2, FL ratio > 64%, and Acute TEVAR are the independent predictors of postoperative AEs in patients with TBAD; (ii) The comprehensive prediction model shows good calibration and reasonable discrimination ability for postoperative AEs in patients with TBAD.

TBAD was characterized by diverse clinical manifestations and rapid disease progression, but effective and timely intervention can significantly reduce mortality. TEVAR have become the primary option over the last decade to address the growing therapeutic need for TBAD^1^. However, there were few studies related to the prognosis of TEVAR, and the existing studies suffered from some limitations, such as a small size of sample, single indicator or simple measurement variable^11–13^, which posed a great challenge to predictive performance. To accurately identify the postoperative AEs for patients with TBAD, we developed a comprehensive prediction model included multimodal data, encompassing the whole procedure details from admission to operation.

The study demonstrated that age > 74 years was an independent risk factors for postoperative AEs, potentially due to the higher occurrence of severe aortic atherosclerosis in the elderly patients. This phenomenon leads to a decreased aortic elasticity and increased brittleness, thereby increasing the risk of aortic dilatation and rupture compared with the young patients. A multi-center study^14^ and Hosn et al.^15^ found that age was an independent risk factor for aortic-related mortality, which is consistent with the results of our study. However, Guido’s^16^ studies indicated that the young patients exhibited an unfavorable outcome, potentially attributed to a higher chance of genetic abnormalities among this population. However, the proportion of patients with TBAD and Marfan syndrome was limited in our study, which may account for the disparity between our findings and those reported in the above study. In any case, more attention should be given to the perioperative management of the elderly patients.

LEM was the most frequently suspected and confirmed malperfusion in our study, presenting with absence of peripheral, motor, or sensory deficit of the lower extremity. The dreaded concern with LEM is the risk of amputation and association with mortality. Bossone et al^17^ reported that aortic dissection with pulse deficits, particularly multiple deficits in either the carotid, branchial, or femoral arteries, had a higher rate of mortality. Charlton-Ouw et al^18^ found that among 104 patients with LEM as a manifested by aortic dissection, 18.3% of them also experienced mesenteric ischemia. Kristofer et al. ^19^ suggested that LEM represents a lager dissection and multiorgan malperfusion. Therefore, we did not exclude the possibility that patients exhibiting clinical symptoms related to LEM in our study might have other malperfusion.

We also found that NT-proBNP > 330 pg/ml is an independent predictor of the occurrence of AEs following TEVAR in patients with TBAD. Although TBAD does not directly affect the ascending aorta, it can still cause malperfusion of the coronary artery. The reasons are as follow: (i) The primary entry tear of patients with TBAD is located at the descending aorta, but the dissection lesion can extend to the aortic arch, which is the portion of the aorta that supplies blood to the coronary artery, preventing them from receiving adequate blood flow; (ii) TBAD can cause changes in decreasing blood pressure and heart rate, which can reduce the perfusion pressure and shorten the perfusion time of the coronary artery, thereby resulting to the coronary artery malperfusion. Luo et al^20^ suggested that NT-proBNP > 210 pg/ml is closely associated with unfavorable outcome in patients with AD. This conclusion may be attributed to the following mechanisms: firstly, coronary artery malperfusion and acute severe pain activate the sympathetic nerve and renin-angiotensin systems (RAS), resulting in elevated blood pressure and left ventricular wall tension, thereby stimulating increased secretion of NT-proBNP^21^; secondly, inflammatory mediators released from the aortic wall directly impacts transcription and translation of NT-proBNP^22^. We conducted a multivariate analysis to control the confounding factors, but did not investigate the risk factors associated with elevated NT-proBNP levels. Further researches are needed to explore the mechanism.

Boufi et al^23^ found that incomplete FL thrombosis and the re-intervention rate after TEVAR were associated with the persistence of distal tears that reverse the blood flow into the FL. Tsai and colleagues^24^ suggested that the connection between the TL and FL exerts an influence on the FL flow volume and pressure, and consequently affects the aortic diameter expansion during the follow-up period. Therefore, we try to cover the distal tears as much as possible during the procedure to prevent the occurrence of re-entry tear. Interestingly, the effective management of distal tears rendered it as a protective factor for postoperative AEs in patients with TBAD. The potential cause may be the distal tears can provide an outlet for blood from the FL, thereby reducing the pressure of FL. This decompression helps to reduce the enlargement of dissection and the risk of aortic rupture. Meanwhile, the research conducted by Anna et al^11^ confirmed our hypothesis, they demonstrated that insufficient outflow, or a mismatch between in- and out-flow, may cause increased pressure of FL. Our findings are also support the therapeutic strategies aimed at intentional increasing outflow to reduce the pressure of FL, such as septal fenestration or complete surgical or endovascular membrane removal. We suggest that the distal tears should be treated more aggressively by employing extended endograft coverage, while carefully balancing the risk of spinal cord ischemia. It is worth trying to improve FL regression with some novel options, including plug application of distal tear or coil embolization of residual FL.

Given the advantages of CTA in the diagnosis, evaluation and postoperative monitoring of patients with TBAD^9,11^, we included several morphological parameters measured on the CTA images into the prediction model. Mendoza and colleagues^25^ reported a discrepancy of 3–5 mm in the measurement of aortic diameter when assessed on axial CT slices. In this study, a dedicated workstation was utilized to measure morphological parameters along the central line of the aorta lumen on the CTA images, and rigorous measurement standards were implemented to minimize potential errors in the measurement work. Prehn et al^26^ found that aortic diameter varies with cardiac cycle, and even during the same cycle, the rate of variation can reach up to 27.5%. Consequently, a single measurement of aortic diameter as a predictor has an inherent limitation. To address this issue, we proposed a novel composite variable of A/D ratio for the first time. The aortic diameter is influenced by various factors, including age, gender, and low-density lipoproteins (LDL). Wolak et al. ^27^ reported an association between the diameter of the ascending aorta and a history of diabetes, while the diameter of the descending aorta was related to smoking. However, we observed no significant differences in terms of diabetes history and smoking between two groups in our study, suggesting that their effects on the A/D ratio can be disregarded. The finding of our study indicates that individuals with TBAD are more likely to develop postoperative AEs when their A/D ratios surpass 1.2. It is worth noting that the A/D ratio refers to the inner diameter of TL, rather than total diameter. The expanded FL compresses the TL, resulting in the overall changes in aortic shape, including increased curvature of the aortic arch and tapering of the TL. These changes will impact the aortic hemodynamics and lead to increased wall shear stress (WSS). All these factors may be associated with the occurrence of AEs following TEVAR^28,29^. Another composite variable in our prediction model is FL ratio. Previous studies have demonstrated the association between FL area and TL perfusion, aortic dilation rate and aortic rupture. The larger FL is, the higher pressure is. While the preoperative distal tears may reduce the acute dilation of FL to some extent.

In our current study, we found Acute TEVAR was one of the predictors of postoperative AEs. On the one hand, patients received TEVAR in the acute phase in our study exhibited more severe complications, including aortic rupture or malperfusion syndrome, which may be the major reason for the more frequent postoperative AEs in these patients. Furthermore, the edematous arterial intima might be fragile and vulnerable to injury from the wire or stent struts at the acute phase, which may lead to the occurrence of RTAD. Whatmore, the edema of dissection flap and aortic wall in the acute phase can indeed affect the accuracy of stent sizing, potentially leading to stent oversizing or undersizing. If the stent does not fit well, it may cause endoleaks. However, the dynamic changes of the intimal flap from acute to chronic phase of aortic dissection, characterized by thickening, straightening, and loss of mobility, may decrease the possibility of the dissection flap to be reapproximated to the aortic wall following TEVAR, thereby making the challenges for elimination of FL and achieving aortic remodeling^30^. Therefore, earlier intervention is potentially advantageous, as the dissection flap is most pliable and provides the best chance for complete remodeling. But this advantage must be balanced with the potential increased risks of damage to the acutely inflamed aorta caused by either the wires or the stent-graft^31^. There seems to be a window of optimal plasticity for the dissection flap to collapse the FL in the subacute phase when the dissection flap is neither so thickened nor so friable, which eventually leads to better long-term outcome while avoiding extra risks in the early phase such as RTAD, SINE, and endoleaks^32^. Several studies^33^ have demonstrated that delayed intervention appears to mitigate the risk of complications associated with TEVAR in patients with stable TBAD. Our study supports a similar perspective that the subacute phase seems to be the best period to perform TEVAR^1^.

In the past year, we developed a program for early telephone follow-up by nurses, an approach that has contributed to identify patients with high-risk signs before an aortic catastrophe occurred. For example, postoperative persistent fever is a readily observable clinical manifestation, and for such patients, we need to alert to the possibility of stent-graft infection, which is a fatal complication. Based on our clinical expertise, antibiotic treatment was commonly employed to patients with persistent fever following TEVAR due to the similarity between infection status and PIS in terms of clinical manifestation. Although our study excluded any potential source of infection before PIS diagnosis, there may still be opportunities for patients with undiagnosed infections to be diagnosed with PIS. Meanwhile, Recent investigations have showed that PIS is an independent predictor of long-term major adverse events (MAEs) (including all cause mortality, aortic rupture, and reintervention) in patient with type B acute aortic syndrome after TEVAR^34^. Therefore, our study suggests that the PIS also needs to be paid adequate attention.

The prediction model demonstrates excellent calibration and reasonable discriminative ability, especially for patients with TBAD who are at the high-risk of developing postoperative AEs. The prediction model will also be used in the future to develop personalized follow-up plans for patients in our medical center. For the high-risk individuals, certain measures can be implemented to prevent postoperative AEs. (i) the preoperative monitoring includes comprehensive evaluation of the patient’s general condition, aortic anatomy, and disease severity, which can help operators and/or surgeons determine the healthy proximal landing zone (PLZ) and select appropriate endovascular devices. (ii) the optimization of the patient’s condition, including controlling blood pressure and heart rate, can help to minimize intraoperative risks. (iii) intraoperative procedures are focus on minimizing aortic wall trauma and ensuring accurate manipulation, deployment, and placement of devices. For example, for the patients with aortic fragility (acute aortic dissection and patients with connective tissue disease), gentle intraoperative procedures and appropriate endograft oversizing is necessary to prevent postoperative AEs. However, it is important to note that despite these efforts, postoperative AEs may still occur due to various factors. Hence, postoperative monitoring, follow-up and implementing timely effective reintervention are equally important for TBAD patients with high-risk signs of postoperative AEs. Our study provides a theoretical foundation for personalized treatment and postoperative monitoring, with the aims of extending the duration of postoperative survival and enhancing the quality of life.

## Limitations

Our study has several limitations. Firstly, the prediction model was developed in a retrospective cohort, which has a relatively small population size. Our cohort only comes from a single center, and only high-quality historic CTA images obtained from June 2017 to June 2021 can be reviewed. Fortunately, single-center research can easily eliminate some confounding factors, such as medication usage and endovascular intervention. Secondly, the model has not been externally verified. We will design multicenter prospective clinical trial to further research and externally verify the prediction model to improve its clinical popularity. Thirdly, our study did not include direct hemodynamics associated with aortic remodeling. We did not consider the hemodynamic parameters associated with different aortic morphologies, we will further refine the collection and analysis of hemodynamic information. At last, the prediction model is based on CTA imaging features of preoperation, and morphological features observed later during follow-up did not be involved. The objective of this study was to identify patients at high risk for AEs following TEVAR in advance, but changes in features over time may introduce new predictive factors that could be incorporated into future research.

## Conclusions

The prediction model developed in our study contributes to the identification of patients with TBAD who are at the high-risk of developing postoperative AEs. For these individuals, meticulous preoperative evaluation and gentle intraoperative procedures can effectively prevent postoperative AEs. Moreover, early detection of AEs is facilitated by shorter follow-up cycle. If externally validated, the prediction model may contribute to development of personalized treatment and postoperative surveillance plans to extend the duration of postoperative survival and enhance the quality of life.

### Supplementary Information

Supplementary Information 1.

Supplementary Information 2.

Supplementary Information 3.

Supplementary Information 4.

Supplementary Information 5.

Supplementary Information 6.

## Data Availability

Data is provided within the manuscript or supplementary information files.
