# Supplementary material for: Predicting adverse events after thoracic endovascular aortic repair for patients with type B aortic dissection
Source: Sci Rep. 2024 Apr 5;14:8057. doi: 10.1038/s41598-024-58106-7 (PMC10997599; doi:10.1038/s41598-024-58106-7)
Supplement: Supplementary file 1 — Supplementary Information 1. [file 41598_2024_58106_MOESM1_ESM.pdf]

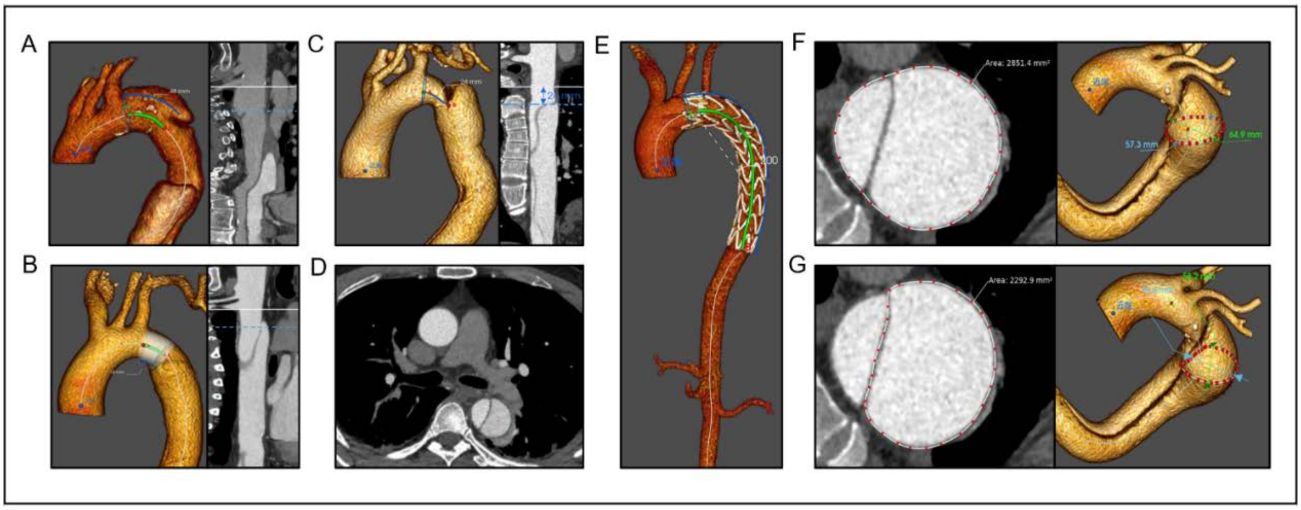

Supplement Fig S1. The measurement method of morphological indicators in this study.

(A-C) The distance from primary entry tear to LSA. The blue line indicates the measurement path of the distance. (A) the internal length (entry tear located at the inner curve of aorta), (B) the external length (entry tear located at the outer curve of aorta), (C) the center line length (entry tear located at the anterior or posterior of aorta). The location of LSA and primary entry tear shown as the white line and the blue dashed line. (D) The measurement section of A/D ratio: pulmonary trunk bifurcation. (E) The length of stent-graft, indicated by the blue line. (F) The area of the total lumen. (G) The area of the maximum FL. The area was enclosed by white line and red dots.
