# Supplementary material for: Predicting adverse events after thoracic endovascular aortic repair for patients with type B aortic dissection
Source: Sci Rep. 2024 Apr 5;14:8057. doi: 10.1038/s41598-024-58106-7 (PMC10997599; doi:10.1038/s41598-024-58106-7)
Supplement: Supplementary file 2 — Supplementary Information 2. [file 41598_2024_58106_MOESM2_ESM.pdf]

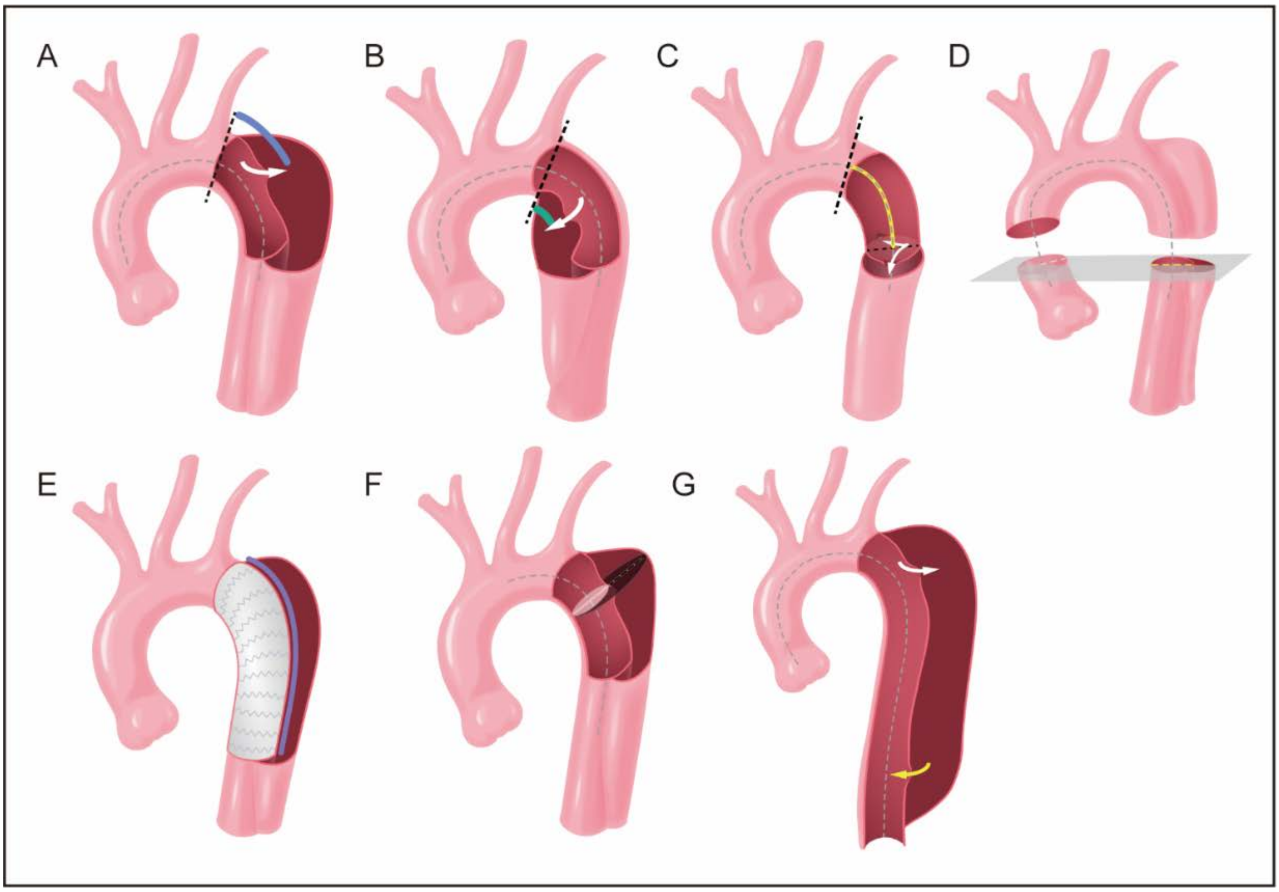

Supplement Fig S2. The schematic diagrams of aortic measurement.

(A-C) The distance from primary entry tear to LSA: the black dotted line indicates the location of LSA, the white arrow marks the location of primary entry tear. The blue, green, and yellow lines represent the measurement paths for different location of primary entry tears. (D) A/D ratio: the grey flat indicates the cross-sectional location of the pulmonary trunk bifurcation, and the white and yellow dotted line represent the TL diameter of ascending and descending aorta, respectively. (E) The measurement paths of stent-graft length, indicated by the purple line. (F) FL ratio: the black flat indicates the area of FL at this section. (G) distal tear, indicated by a yellow arrow.
