# Supplementary figures and images for: Predicting adverse events after thoracic endovascular aortic repair for patients with type B aortic dissection
Source: Sci Rep. 2024 Apr 5;14:8057. doi: 10.1038/s41598-024-58106-7 (PMC10997599; doi:10.1038/s41598-024-58106-7)

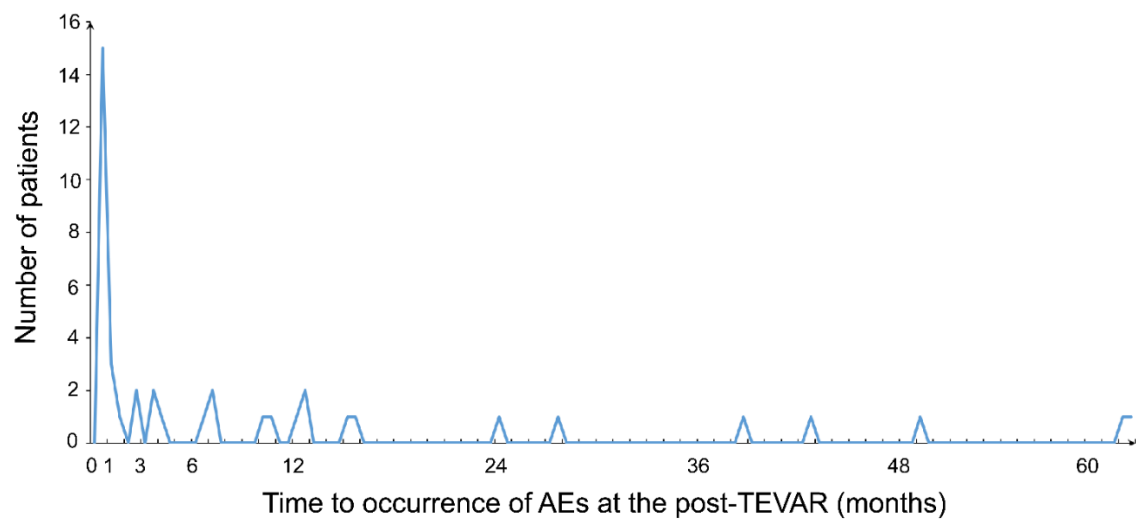

**Supplement Fig S3.** The duration of AEs following TEVAR in patients of our study.

Supplement: Supplementary file 3 — Supplementary Information 3. [file 41598_2024_58106_MOESM3_ESM.pdf]

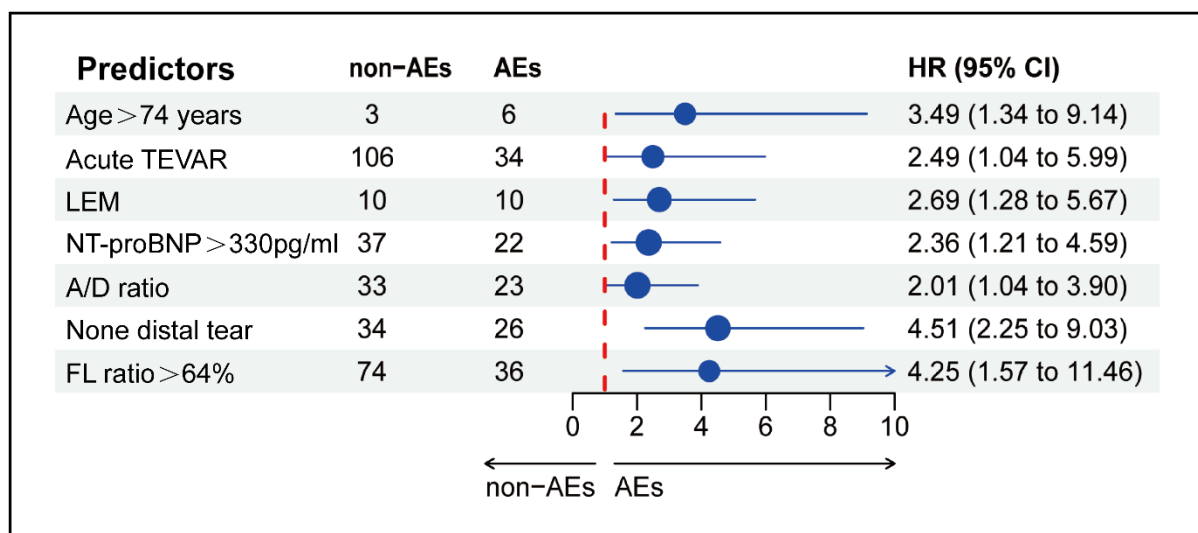

**Supplement Fig S4.** The forest plot of the multivariate COX regression analyses.

Supplement: Supplementary file 4 — Supplementary Information 4. [file 41598_2024_58106_MOESM4_ESM.pdf]
