# Supplementary material for: Predicting adverse events after thoracic endovascular aortic repair for patients with type B aortic dissection
Source: Sci Rep. 2024 Apr 5;14:8057. doi: 10.1038/s41598-024-58106-7 (PMC10997599; doi:10.1038/s41598-024-58106-7)
Supplement: Supplementary file 5 — Supplementary Information 5. [file 41598_2024_58106_MOESM5_ESM.pdf]

**Supplement Table S1.** The univariate analysis of the baseline characteristics.

| Variables | Regression Coefficient | HR (95% CI) | *P* |
|---|---|---|---|
| **Age(years)** | 0.00 | 1.000 (0.973-1.028) | 0.989 |
| **Age⩾74 years, n (%)** | 1.322 | 3.751 (1.568-8.972) | **0.003** |
| **Male, n (%)** | 0.297 | 1.346 (0.596-3.036) | 0.474 |
| **SBP, mmHg** | -0.005 | 0.995 (0.985-1.005) | 0.342 |
| **DBP, mmHg** | -0.007 | 0.993 (0.997-1.010) | 0.432 |
| **HR, time/min** | -0.004 | 0.996 (0.974-1.018) | 0.715 |
| **Sudden chest pain, n (%)** | -0.466 | 0.628 (0.329-1.198) | 0.158 |
| **Myocardial ischemia, n (%)** | -0.278 | 0.757 (0.396-1.448) | 0.4 |
| **Pericardial effusion, n (%)** | 0.166 | 1.181 (0.364-3.834) | 0.782 |
| **Pleural effusion, n (%)** | -0.006 | 0.994 (0.515-1.920) | 0.986 |
| **LEM, n (%)** | 1.163 | 3.199 (1.566-6.535) | **0.001** |
| **Hypertension, n (%)** | -0.734 | 0.480 (0.255-0.903) | **0.023** |
| **History of TAA, n (%)** | 0.804 | 2.234 (0.539-9.269) | 0.268 |
| **Marfan syndrome, n (%)** | 0.853 | 2.347 (0.322-17.127) | 0.4 |
| **TAI, n (%)** | -0.395 | 0.674 (0.092-4.912) | 0.697 |
| **History of cardiac operation, n (%)** | 1.012 | 2.752 (0.661-11.453) | 0.164 |
| **Diabetes, n (%)** | -0.493 | 0.611 (0.084-4.452) | 0.627 |
| **CAD, n (%)** | -0.096 | 0.909 (0.323-2.552) | 0.856 |
| **Stroke, n (%)** | 0.356 | 1.428 (0.600-3.401) | 0.421 |
| **COPD, n (%)** | 0.064 | 1.067 (0.379-2.999) | 0.903 |
| **Cancer, n (%)** | 0.617 | 1.854 (0.446-7.701) | 0.396 |
| **Smoker, n (%)** | 0.464 | 1.590 (0.842-3.003) | 0.153 |
| **Drinker, n (%)** | -0.461 | 0.631 (0.194-2.048) | 0.443 |
| **Hb, g/L** | -0.005 | 0.995 (0.982-1.009) | 0.517 |
| **PLT, ×10$^9$/L** | -0.001 | 0.999 (0.994-1.004) | 0.596 |
| **WBC, ×10$^9$/L** | -0.17 | 0.983 (0.900-1.073) | 0.701 |
| **NLR, %** | -0.002 | 0.998 (0.986-1.010) | 0.747 |
| **CRP, mg/L** | -0.003 | 0.997 (0.987-1.007) | 0.52 |
| **ALT, U/L** | -0.003 | 0.997 (0.985-1.010) | 0.654 |
| **Scr, μmol/L** | 0.001 | 1.001 (1.000-1.003) | 0.12 |
| **D-dimer, mg/L** | 0.011 | 1.011 (0.980-1.043) | 0.484 |
| **NT-proBNP, pg/mL** | 0 | 1.000 (1.000-1.000) | **0.047** |
| **NT-proBNP＞330 pg/mL, n(%)** | 0.851 | 2.343 (1.268-4.330) | **0.007** |
| **CK, U/L** | 0 | 1.000 (1.000-1.001) | **0.008** |

Values are expressed as mean ± SD or n (%), SD, standard deviation.

Significant values are in bold.

HR, hazard ratio; CI, confidence interval; SBP, systolic blood pressure; DBP, diastolic blood pressure; HR: heart rate; LEM, lower extremity malperfusion; TAA, thoracic aortic aneurysm; TAI, traumatic aortic injury; CAD, coronary artery disease; COPD, chronic obstructive pulmonary disease; Hb, hemoglobin; PLT, platelet; WBC, white blood cell; NLR, neutrophil-to-lymphocyte ratio; CRP, C-reactive protein; ALT, alanine aminotransferase; Scr, serum creatinine; NT-proBNP, N-terminal pro-B-type natriuretic peptide; CK, creatine kinase.
