# Supplementary material for: Predicting adverse events after thoracic endovascular aortic repair for patients with type B aortic dissection
Source: Sci Rep. 2024 Apr 5;14:8057. doi: 10.1038/s41598-024-58106-7 (PMC10997599; doi:10.1038/s41598-024-58106-7)
Supplement: Supplementary file 6 — Supplementary Information 6. [file 41598_2024_58106_MOESM6_ESM.pdf]

Supplement Table 2 The univariate analysis of the baseline CTA imaging characteristics, the intraoperative conditions and stent-graft information.

Variables	Regression Coefficient	HR (95% CI)	*P*
**The baseline CTA imaging characteristics**			
**The aortic diameter of the proximal end of the LSA, mm**	-0.084	0.919 (0.816-1.036）	0.166
**The aortic diameter of the tracheal bifurcation, mm**	0.011	1.011 (0.967-1.057)	0.632
**The aortic diameter of the tracheal bifurcation＞40mm, n (%)**	0.667	1.948 (1.021-3.716)	**0.043**
**The ascending aortic diameter, mm**	-0.027	0.973 (0.898-1.055)	0.508
**The descending aortic diameter, mm**	0.01	1.010 (0.970-1.051)	0.627
**The A/D ratio**	2.892	18.037 (4.761-68.340)	＜**0.001**
**The A/D ratio＞1.2, n (%)**	1.166	3.210 (1.730-5.957)	＜**0.001**
**The maximum descending aortic diameter, mm**	0.017	1.017 (0.983-1.053)	0.329
**The diameter of the primary entry tear, mm**	0.04	1.041 (1.016-1.067)	＜**0.001**
**The diameter of the primary entry tear＞12.8mm, n (%)**	1.062	2.892 (1.543-5.422)	＜**0.001**
**The location of the primary entry tear (inner curvature), n (%)**	0.147	1.158 (0.535-2.508)	0.71
**The median distance from the primary entry tear to the LSA, mm**	-0.004	0.996 (0.986-1.006)	0.414
**None distal tear, n (%)**	1.376	3.957 (2.094-7.480)	＜**0.001**
**The length of the thoracic aorta, mm**	0.001	1.001 (0.993-1.009)	0.783
**The length of stent graft, mm**	0.005	1.005 (0.999-1.012)	0.085
**Maximum area of the total aortic lumen, mm²**	0	1.000 (1.000-1.001)	0.427
**Maximum area of the false lumen, mm²**	0	1.000 (1.000-1.001)	0.149
**The FL ratio, %**	0.031	1.031 (1.010-1.053)	**0.004**
**The FL ratio＞64%, n (%)**	1.471	4.355 (1.708-11.104)	**0.002**
**The branch arteries involvement, n (%)**	0.125	1.133 (0.608-2.112)	0.693
**The intraoperative conditions and stent-graft information**			
**Acute TEVAR, n (%)**	-0.255	0.775 (0.343-1.751)	0.54
**Stent graft passage (LFA), n (%)**	-0.53	0.589 (0.300-1.154)	0.123
**LSA coverage, n (%)**			
**0%**	/	/	0.190
**25%**	0.082	1.086 (0.251-4.688)	0.912
**50%**	0.524	1.689 (0.152-18.764)	0.669
**75%**	0.183	1.201 (0.241-5.989)	0.823
**100%**	1.868	6.475 (0.884-47.431)	0.066
**LSA revascularization techniques**			
**Branched stent-graft, n (%)**	-0.605	0.546 (0.213-1.400)	0.208
**Chimney technique, n (%)**	0.173	1.189 (0.594-2.378)	0.625
**Fenestration, n (%)**	0.707	2.028 (0.488-8.427)	0.331
**Bank of stent-graft, n (%)**			
**1**	/	/	0.560
**2**	-0.584	0.558 (0.218-1.426)	0.223
**3**	-0.176	0.3441 (0.136-0.856)	**0.022**
**The distal covered stenting, n (%)**	0.484	1.622 (0.875-3.006)	0.124
**TSG, n (%)**	-0.066	0.936 (0.473-1.852)	0.85
**Operator, n (%)**			
**A**	/	/	0.560
**B**	-0.584	0.558 (0.218-1.426)	0.223
**C**	-0.176	0.3441 (0.136-0.856)	**0.022**
**Intraoperative endoleak, n (%)**	0.462	1.588 (0.700-3.599)	0.268

Values are expressed as mean ± SD or n (%), SD, standard deviation.

Significant values are in bold.

HR, hazard ratio; CI, confidence interval; LSA, left subclavian artery; A/D ratio, the ratio of the diameter of the ascending and descending aorta; FL, false lumen. TEVAR; thoracic endovascular aortic repair; LFA, left femoral artery; LSA, left subclavian artery; TSG; tapered stent-graft.
